# Parvovirus Infection-Related Anemia after Kidney Transplantation

**DOI:** 10.1155/2020/6437392

**Published:** 2020-01-31

**Authors:** Neeraj Sharma, Ranvir Bajwa

**Affiliations:** ^1^University of Southern California, Department of Medicine, Division of Nephrology, Los Angeles, California, USA; ^2^Keck School of Medicine of USC, 1975 Zonal Ave., Los Angeles, CA 90033, USA

## Abstract

Anemia postkidney transplantation is an important issue which has been correlated with increased hospitalizations and higher mortality. Infections, including those due to parvovirus B19, CMV, and BK virus (polyomavirus), have also been associated with an increased risk of anemia. Here, we present a case of new-onset anemia in a kidney transplant recipient within 3 months of transplant. The patient was found to have multiple viral infections from parvo B19, BK virus, and rhinovirus. The anemia resolved completely after successful reduction in the parvo B19 and BK viral load. Workup for viral infections must be considered in the differential diagnosis of postkidney transplant anemia.

## 1. Introduction

Posttransplant anemia is estimated to occur in 20-50% of patients depending on the criteria used to define anemia. In patients with well-functioning renal allografts, anemia usually resolves by 3 to 6 months posttransplant [1]. The development of early posttransplant anemia is likely a multifactorial process, and the identification of the underlying cause is vital in choosing the appropriate therapy [2]. The most common causes of early posttransplant anemia can be divided into three categories: inadequate erythropoietin, iron deficiency, or decreased erythropoiesis. The latter is most commonly attributed to bone marrow suppression from immunosuppressive therapy or infections, particularly from viruses.

Many viruses, including parvovirus B19, cytomegalovirus, BK virus, Epstein-Barr virus (EBV), hepatitis B, hepatitis C, and HIV, can interfere with myelopoiesis and cause aplastic anemia [3]. Parvovirus B19 infection is a cause of acute anemia following renal transplantation. Although parvovirus B19 is likely the main pathogen in pathogenesis of anemia, coinfections with other viruses may worsen the degree of anemia. Parvovirus B19-related anemia has been reported in solid-organ transplantation; however, there have not been any case reports describing dual viral infections with parvovirus B19 and BK leading to acute anemia.

## 2. Case Presentation

A 76-year-old Hispanic female with hypertension and ESRD on maintenance hemodialysis for six years underwent a deceased donor kidney transplant in November 2018. The donor was a non-DCD, 44-year-old female with KDPI of 77%. HLA mismatching showed 2 mismatches at A locus, 2 mismatches at B locus, and 2 mismatches at DR locus. The patient had a calculated PRA of zero percent. T-flow crossmatch was 69 and 55 after pronase. Cold ischemia time was 10 hours 33 minutes. The patient received induction with antithymocyte globulin (ATG) with a total dose of 3 mg/kg. There was delayed graft function, and the patient had HD on POD 1, 2, 4, and 10. She also received 1-unit PRBC on POD 1. The patient was discharged on POD 4 with maintenance immunosuppression consisting of tacrolimus, mycophenolate mofetil, and prednisone. The ureteral stent was removed 4 weeks postop. On POD 10, the patient developed de novo DSA to HLA-C and DQB1 with MFI 1,529 and 1,855, respectively. Immunosuppression was intensified, and tacrolimus trough levels were maintained between 8 and 10 one month posttransplant. Graft function began to improve on POD 10 and eventually normalized at serum creatinine of 0.9 mg/dl.

During the second month posttransplant, the patient developed BK viremia with viral load copies of 2,413 for which mycophenolate mofetil was reduced from 1 gm twice daily to 250 mg twice daily. DSA were rechecked which remained unchanged. During this time, the hemoglobin level decreased to 9.2 g/dl from baseline 12 g/dl. One month later, anemia had worsened significantly to 8.1 g/dl with MCV of 105.2 fl causing fatigue and dyspnea on exertion. A repeat serum BK virus PCR revealed increasing copies of BK virus up to 6,234 copies. The patient was also found to have rhinovirus detected in the nasal swab. A posttransplant anemia workup was performed. Acute rejection or AKI was unlikely as the patient had normal kidney function. There was no evidence of gastrointestinal or genitourinary bleeding. Nutritional deficiencies as well as hypothyroidism were ruled out. Drug-related anemia was suspected. However, despite discontinuation of TMP-SMZ and valganciclovir, there was no improvement in Hgb. In addition, mycophenolate mofetil was reduced as well without significant improvement. As a result of the worsening anemia, a qualitative RT-polymerase chain reaction (PCR) for parvovirus B19 was sent which revealed greater than 100 million copies. Other laboratory studies showed hematocrit 26.2% (35-47), platelets 325 mm^3^ (150-400), leukocyte count 3.15 mm^3^ (4.1-10.9), reticulocyte relative 19.3% (0.5-2.0), d-dimer 3,908 ng/ml (<240), fibrinogen 380 mg/dl (100-800), haptoglobin < 10 mg/dl (30-200), LDH 353 *μ*/l (135-214), transferrin saturation 37% (15-50), ferritin 1,937 ng/ml (5-204), B12 520 pg/ml (252-1245), and folate 9.0 ng/ml (>4.8).

A bone marrow biopsy was deferred as the worsening anemia was attributed to parvovirus given the strongly elevated copies of greater than 100 million copies/ml. In addition, concomitant viral infections from BK virus and rhinovirus likely further worsened the degree of anemia.

**Figure 1:**
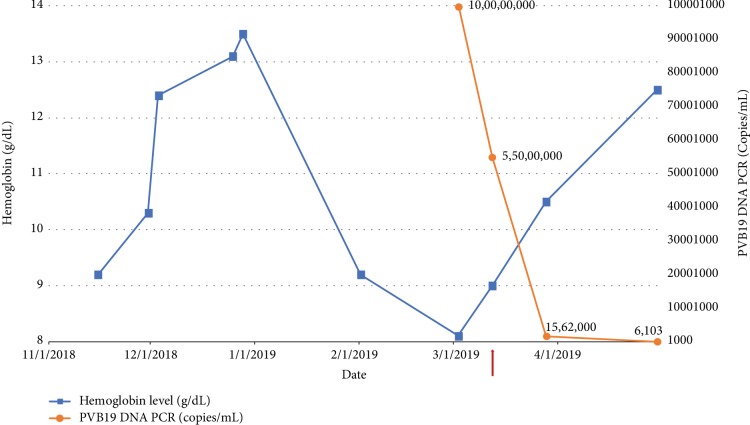
Time course of hemoglobin (blue line and circles) with parvovirus B19 levels (red line and circle). Administration of IVIG is shown by the arrow. IVIG was given as 500 mg/kg/day weekly over 5 sessions. In addition, the patient also received one dose of cidofovir the day after first IVIG therapy for treatment of BK viremia.

The patient was started on intravenous immunoglobulin (IVIG) at a dose of 500 mg/kg/day weekly for a total of 5 treatments. Due to the concomitant BK viremia, the patient received one dose of cidofovir 0.5 mg/kg. After one session of IVIG, the hemoglobin rose to 9.0 g/dl. After a total of 5 sessions of IVIG, the hemoglobin level further increased to 13.1 g/dl with improvement in symptoms. Repeat parvo B19 PCR levels were reduced to 8,537 copies, and serum BK virus PCR also improved to 510 copies (Figure 1). Single antigen bead testing revealed persistent donor-specific antibodies to class I and multiple class II antigens with MFIs ranging between 1,000 and 2,000. The patient has been maintained on low-dose mycophenolate mofetil in addition to lower tacrolimus trough levels between 4 and 6 ng/ml. The patient has well-preserved allograft function and continues to follow up regularly in the postkidney transplant clinic.

## 3. Discussion

This is one of the very few reported cases of concurrent parvovirus B19 and BK viremia-related acute early postkidney transplant anemia. Our patient, who had normal hemoglobin levels one month posttransplant, later developed an acute drop in hemoglobin levels approximately three months after kidney transplantation. Initial laboratory studies revealed reticulocytosis which was due to low-grade hemolysis which confounded our suspicion of an infection-related anemia. Furthermore, macrocytosis is usually not seen in parvovirus-related anemia, but we believe that this may be falsely elevated given the high reticulocyte count. Since our patient did not have a response to packed cell transfusion or epoetin alfa, a quantitative PCR for serum parvovirus B19 DNA was sent which was strongly positive at greater than 100 million copies. The etiology of the low-grade hemolysis remains unexplained, and this may have contributed to the delayed diagnosis of parvoviremia. Nevertheless, our patient's anemia resolved completely after therapy with IVIG and reduction of immunosuppression.

It remains unclear whether it was the parvovirus or polyomavirus that led to anemia. Little is known about the mechanism on how BK infection causes anemia. However, in one report [4] describing the mechanism of myeloproliferative disease caused by polyomavirus in mice infected with BKV, the authors hypothesized that anemia was due to production of cytokines and growth factors by BKV-infected cells causing polyclonal proliferation of one or more cell types of the hematopoietic system.

We suspect that the anemia was driven primarily as a result of parvovirus infection since plasma polyomavirus PCR has only BK virus 6,234 copies which is unlikely to cause kidney dysfunction or any urological conditions. Little is known about the mechanisms by which viruses cause hematological abnormalities. However, several possibilities [5] have been suggested which include production of inhibitory cytokines which alter cell function and decrease production of hematopoietic factors, as well as direct infection of hematopoietic progenitor cells.

A few cases of parvovirus-related anemia postkidney transplant have been reported. Pakkyara et al. [6] reported a case of a bone marrow biopsy proven parvovirus B19 infection. IVIG was found to be beneficial. Ahmed et al. [7] reported 2 cases of severe anemia secondary to parvovirus B19 infection. Both cases described recovery of the hemoglobin level after IVIG therapy. In reviewing, these cases do not describe concurrent infection with other viruses such as in our patient.

In an excellent review of 98 cases by Eid et al. [8], it showed that the use of PCR for diagnosis is helpful in immunosuppressed patients since they fail to mount antibodies against parvovirus B19 during active infection. Their study showed that a parvovirus B19 IgM serologic test was negative in 29% of patients. Hence, a negative parvovirus B19 IgM antibody test does not rule out diagnosis and may delay treatment in patients at centers who rely on serological examination for parvovirus B19 diagnosis. If serology and PCR are negative but the clinical suspicion remains high, then a bone marrow biopsy would be helpful in establishing the diagnosis.

Kidney transplant recipients may acquire parvovirus B19 infection through the transplant graft or by reactivation of an endogenous latent or persistent infection following immunosuppression [9]. A retrospective review by Capenko et al. [10] showed a statistically significant correlation between parvovirus B19 infection and anemia. The median time to the onset of anemia after transplantation is seven weeks [6].

Parvovirus B19 replicates in erythroid precursors and causes RBC aplasia by inducing RBC precursor apoptosis [11]. The virus encodes two structural proteins, viral protein (VP1) and viral protein (VP2) and a nonstructural protein (NS1). It has been well described that the virus binds to the blood group P-antigen, which is expressed abundantly on erythroblasts. After binding to P-antigen, the virus causes apoptosis of the erythrocyte progenitors via the (nonstructural) NS1 protein-caspase pathway [12].

Baek et al. [13] showed that nearly 90% of positive cases were reported within the first year after kidney transplantation. The major risk factor for parvovirus B19 infection is immunosuppression, which is shown due to resolution of anemia when immunosuppression is lowered [14]. Furthermore, it has been shown that induction therapy with antithymocyte globulin has a high risk for parvovirus B19 infection compared with Basiliximab [15].

The first-line treatment for parvoviremia should be reduction in immunosuppression, as this would allow the immune system to mount specific immunity against parvovirus B19. This observation leads to the current practice of IVIG therapy for parvovirus B19 treatment [16]. IVIG contains antibodies to a number of potential pathogens, which may provide a level of passive immunity leading to the successful treatment of posttransplantation viral infections, including parvovirus B19, BK virus, and Epstein-Barr virus (EBV) [17]. In conclusion, concomitant viral infections can complicate parvovirus-related anemia in renal transplant patients. As a result, this emphasizes the importance of early screening and treatment of this potentially life-threatening complication.
